# The Role of Cow’s Milk Consumption in Breast Cancer Initiation and Progression

**DOI:** 10.1007/s13668-023-00457-0

**Published:** 2023-02-02

**Authors:** Bodo C. Melnik, Swen Malte John, Pedro Carrera-Bastos, Loren Cordain, Claus Leitzmann, Ralf Weiskirchen, Gerd Schmitz

**Affiliations:** 1 Department of Dermatology, Environmental Medicine and Health Theory, University of Osnabrück, D‐49076 Osnabrück, Germany; 2 Institute for Interdisciplinary Dermatological Prevention and Rehabilitation (iDerm) at the University of Osnabrück, Lower-Saxonian Institute of Occupational Dermatology (NIB), Osnabrück, Germany; 3 Center for Primary Health Care Research, Lund University/Region Skåne, Skåne University Hospital, 205 02 Malmö, Sweden; 4 Faculty of Biomedical and Health Sciences, Universidad Europea de Madrid, 28670 Madrid, Spain; 5 Centro de Estudios Avanzados en Nutrición (CEAN), 11007 Cádiz, Spain; 6 Colorado State University, Fort Collins, CO USA; 7 Institute of Nutrition, University of Giessen, 35390 Giessen, Germany; 8 Institute of Molecular Pathobiochemistry, Experimental Gene Therapy and Clinical Chemistry (IFMPEGKC), RWTH University Hospital Aachen, D‐52074 Aachen, Germany; 9 Institute for Clinical Chemistry and Laboratory Medicine, University Hospital of Regensburg, University of Regensburg, D‐93053 Regensburg, Germany

**Keywords:** BRCA1, Breast cancer, Cow’s milk consumption, Estrogens, Exosomal microRNAs, Fat mass and obesity-associated gene, Insulin-like growth factor 1

## Abstract

**Purpose of Review:**

This review evaluates cow milk’s impact on breast carcinogenesis by linking recent epidemiological evidence and new insights into the molecular signaling of milk and its constituents in breast cancer (BCa) pathogenesis.

**Recent Findings:**

Recent prospective cohort studies support the association between cow’s milk consumption and the risk of estrogen receptor-*α*-positive (ER^+^) BCa. Milk is a complex biological fluid that increases systemic insulin-like growth factor 1 (IGF-1), insulin and estrogen signaling, and interacting hormonal promoters of BCa. Further potential oncogenic components of commercial milk include exosomal microRNAs (miR-148a-3p, miR-21-5p), bovine meat and milk factors, aflatoxin M1, bisphenol A, pesticides, and micro- and nanoplastics. Individuals with *BRCA1* loss-of-function mutations and *FTO* and *IGF1* gain-of-function polymorphisms enhancing IGF-1/mTORC1 signaling may be at increased risk for milk-induced ER^+^ BCa.

**Summary:**

Recent prospective epidemiological and pathobiochemical studies identify commercial milk consumption as a critical risk factor of ER^+^ BCa. Large meta-analyses gathering individuals of different ethnic origins with milk derived from dairy cows of varying genetic backgrounds and diverse feeding procedures as well as missing data on thermal processing of milk (pasteurization versus ultra-heat treatment) make multi-national meta-analyses unsuitable for BCa risk estimations in susceptible populations. Future studies are required that consider all vulnerable periods of breast carcinogenesis to cow’s milk exposure, beginning during the perinatal period and puberty, since these are the most critical periods of mammary gland morphogenesis. Notwithstanding the need for better studies including detailed information on milk processing and vulnerable periods of human breast carcinogenesis, the available evidence suggests that dietary guidelines on milk consumption may have to be reconsidered.

## Introduction

In 2020, 2.3 million women were diagnosed with breast cancer (BCa) with 685,000 deaths globally. At the end of 2020, 7.8 million were diagnosed with BCa in the past 5 years, making BCa the world’s most prevalent cancer [1]. The estimated new total BCa cases in the United States (US) for 2022 are 287,750 for females and 2710 for males, respectively [2]. BCa prevalence is high in industrialized countries, where cow’s milk and dairy consumption are major dietary components. From 1947 to 1997, the age-standardized death rate of BCa in Japan increased about 2-fold, and the respective intake of milk increased 20-fold [3]. This review aims to interpret the latest epidemiology in context with recent insights into the molecular signaling of milk linking cow’s milk consumption and BCa risk.

Milk is not a simple dietary food, but a growth-promoting endocrine system enhancing the synthesis of insulin-like growth factor-1 (IGF-1) [4], which activates the nutrient- and growth factor-sensitive kinase mechanistic target of rapamycin complex 1 (mTORC1) [5]. Physiologically, milk signaling is restricted to the period of lactation in all mammals except Neolithic and modern humans, who may be persistently exposed to cow’s milk. And even in societies who adopted dairy early on, milk was preferentially consumed in fermented forms until the widespread implementation of pasteurization and refrigeration technology [6]. In industrialized countries, cow’s milk intake in non-fermented forms may be an exposure over lifetime, beginning with maternal cow’s milk consumption during pregnancy, continued by cow’s milk intake in infancy, childhood, adolescence, and pre- and postmenopausal life [7•], which, as it will be discussed below, may influence BCa risk, particularly during certain vulnerable periods involved in breast carcinogenesis.

## Search Strategy and Selection Criteria

PubMed was searched for original research articles, retrospective and prospective cohort studies, case-control studies, and meta-analyses/systematic reviews conducted in humans over the last 5 years relating cow’s milk consumption to the risk of BCa in humans. Search terms included “cow milk,” “dairy,” “diet,” “milk,” “non-fermented milk,” “fermented milk,” “breast cancer,” “mammary tumor,” and “breast cancer risk.” Milk-related compounds like “insulin-like growth factor 1,” “estrogens,” “bovine meat and milk factors,” “aflatoxins,” “bisphenol A,” “pesticides,” “microplastics,” and “nanoplastics” were linked to known pathogenic pathways in breast carcinogenesis. Factors associated with BCa risk including “birthweight,” “menarche,” “body mass index” “juvenile myopia,” “acne vulgaris,” and “linear growth” were also considered.

## Increased Fetal Growth and Birthweight

Humans are the only mammalian species consuming the milk of another mammal during pregnancy, recommended by health professionals and dietary guidelines because milk is a rich source of calcium and vitamin D, the latter in countries with vitamin D fortification schemes. Milk contains a moderate amount of protein composed of essential branched-chain amino acids (BCAAs) thought to have beneficial effects for nutrition during pregnancy. The Generation R Study, a population-based prospective cohort study from fetal life until young adulthood in Rotterdam, investigated 3405 mothers during pregnancy [8]. Maternal cow’s milk consumption of > 3 glasses (450 ml of milk) per day was associated with greater fetal weight gain in the third trimester of pregnancy [8]. Worldwide studies confirmed an increase in fetal growth and birthweight in relation to milk consumption during pregnancy [9, 10]. Compared to women who had a normal birthweight (2500–3999 g), women who weighed ≥ 4000 g at birth had a 20 percent to fivefold increased risk of premenopausal BCa [11]. Birthweight is positively associated with BCa risk [12] as well as mammographic density among postmenopausal and less among premenopausal women [13].

Cow’s milk consumption enhances growth hormone (GH) levels in children and peak GH levels in adults [14, 15], as well as circulating IGF-1 levels in children and adults [4, 14–18, 19••, 20•]. There are two mechanisms leading to milk-mediated elevations of circulatory IGF-1 levels of the milk recipient: (1) uncertain proportions of bovine milk IGF-1, which shares an identical amino acid sequence with human IGF-1 [21], may be absorbed in the human intestine. (2) milk components, especially milk protein-derived amino acids (Trp, Arg, Met), may induce the synthesis and secretion of pituitary GH and hepatic IGF-1 into the circulation [5, 7•].

Of note, the administration of IGF-1 to pregnant mice resulted in significantly heavier birth and postnatal bodyweights of the offspring when compared to untreated controls. Morphometric analyses revealed that a prenatal dose of 5 μg IGF-1 resulted in significantly longer ductal elongation and higher breast density in the offspring. Furthermore, 5 μg IGF-1 also resulted in the highest number of breast stem/progenitor cells in the offspring when compared to controls whose mothers were not treated with IGF-1 during pregnancy [22]. These findings provide evidence for a prenatal IGF-1-mediated modulation of breast stem cell composition and breast density in the offspring [22]. Thus, the GH/IGF-1 axis may play an important role in regulating breast stem cell numbers during a prenatal developmental window [23].

## Breastfeeding Versus Artificial Formula Feeding

For infants and nursing women, breastfeeding provides protection against BCa [24–26]. An inverse association was found between an increase in body mass index (BMI) and the duration of exclusive breastfeeding (EXBF) among carriers of the risk allele of the fat mass and obesity-associated gene (*FTO*) rs9939609 [27]. EXBF antagonizes the FTO rs9939609 risk allele and by the age of 15 years, the predicted reduction in BMI after 5 months of EXBF was 0.56 kg/m^2^ (95% CI: 0.11–1.01; *p* = 0.003) and 1.14 kg/m^2^ (95% CI: 0.67–1.62; *p* < 0.0001) in boys and girls, respectively [28]. Compared to infants, who received EXBF, FTO levels in blood mononuclear cells of infants fed artificial formula were excessively overexpressed [29••]. Notably, infant formula is deficient in human milk exosomes and microRNAs (miRs) including miR-30b [30••], which targets and suppresses *FTO* expression [31]. FTO is an N6-methyladenosine (m6A) demethylase and participates in the epigenetic regulation of adipogenesis and tumorigenesis thus changing mRNA expression networks and through interaction with mTORC1 [32, 33]. Similar molecular mechanisms play a role in the development of obesity and BCa [34, 35•], which exhibit overexpression of FTO [36]. Thus, the nutrigenomic and epigenetic regulation of FTO during postnatal life appears to be a critical window for breast carcinogenesis.

## Early-Life BMI, Menarche, and Thelarche

BMI is critically related to the initiation of puberty. In concordance with total and percentage body fat, all pubertal stages began earlier in females with BMI ≥ 85th percentile comparable to females with average BMI [37]. The National Health and Nutrition Examination Survey (NHANES) observed that among children 5–10 years of age, those in the highest quartile (Q-IV) for milk intake had higher BMIs than those in lower Q-II [38]. Of note, milk had more consistent positive associations with BMI than did any other dairy product [38]. Every 5 kg/m^2^ increase in early-life BMI was associated with an elevated risk of BCa in a recent dose–response meta-analysis [39]. A recent systematic review and meta-analysis of cross-sectional and prospective cohort studies assessed the associations between total dairy consumption and its different subtypes with the prevalence and incidence of overweight, obesity, and overweight/obesity in children and adolescents [40]. Regarding prospective studies, total milk consumption was positively associated with overweight prevalence (OR (95% CI): 1.13 (1.01–1.26)) and incidence (RR (95%CI): 1.17 (1.01–1.35)) risk [40].

The NHANES and the Tehran Lipid and Glucose Study reported an association between cow’s milk consumption and early onset of menarche [41, 42], a further recognized risk factor for BCa [43], which correlates with breast density [44]. When considering early thelarche (< 10 years) and early menarche (< 12 years) together, women with both had a 30% higher risk of BCa compared with women with neither risk factor [45]. IGF-1 plays a crucial role in hypothalamic-pituitary-ovarian hormone-controlled metabolic processes that influence the onset of menarche [46]. In fact, serum levels of IGF-1 increase with age and pubertal development [47]. Noticeably, a more frequent consumption of milk-based drinks, which may increase circulating IGF-1 levels [4], was associated with a higher percentage of fibroglandular volume (FGV) measured at Tanner stage 4, whereas higher yogurt intake was associated with a lower FGV and delayed age at menarche in Chilean girls [48]. Earlier age of menarche promoted by cow’s milk consumption but not fermented dairy products may thus enhance the susceptibility to breast carcinogenesis during prepuberty and puberty.

## Longitudinal Growth During Puberty

There is a well-established relationship between cow’s milk consumption during childhood and linear growth-enhancing height [49–51]. The consumption of milk, but not other dairy products, was associated with height among US preschool children in the NHANES 1999–2002 [49], whereas cow’s milk consumption was a significant predictor of the height of 12–18-year old adolescents [50]. The mitogen IGF-1 is the major inducer of bone growth [51–54]. During puberty, circulating IGF-1 promotes bone periosteal apposition [53]. Girls with higher serum IGF-1 levels in childhood enter puberty earlier [54]. Pubertal timing is influenced by IGF-1 promoting longitudinal growth earlier in childhood [54].

Biro et al. [55••] demonstrated that peak height velocity (PHV) was greatest in early, and least in late-maturing girls. The length of the pubertal growth spurt was longest in early, and shortest in late-maturing girls. Earlier onset of menarche was related to greater PHV. IGF-1 concentrations were tracked significantly during puberty and higher IGF-1 was related to earlier age of PHV, earlier age of menarche, greater PHV, and taller adult height [55••].

## Acne and Juvenile-Onset Myopia–Indicators of Excessive IGF-1 Signaling

A visible indicator disease of exaggerated IGF-1 signaling is acne vulgaris, the most common inflammatory skin disease in industrialized countries, which is associated with increased height and BMI during puberty [56–58]. The consumption of dairy foods, particularly milk, and high glycemic carbohydrates, a common dietary pattern seen in acne patients of Westernized populations [59], increases circulatory levels of IGF-1 and insulin [60–63]. These findings point to accelerated IGF-1-mediated growth trajectories in acne pathogenesis leading to the hyperproliferation of sebaceous glands promoted by milk consumption [64–66]. In contrast, individuals with Laron syndrome exhibiting severe congenital IGF-1 deficiency do not develop acne vulgaris and are of small stature [67]. They are protected from common cancers including BCa [68]. Remarkably, the Sister Study recently showed that ever being diagnosed with severe acne before the age of 18 years was associated with a higher risk of BCa [69•].

Juvenile-onset myopia caused by increased vitreal chamber growth is another IGF-1-induced condition also related to increased height and BMI during adolescence [70]. According to a cross-sectional study of children 6–12 years in China, breastfeeding was associated with a decreased risk of myopia [71].

## IGF-1 and Pubertal Mammary Gland Morphogenesis

Mammary development occurs almost entirely during puberty [72]. IGF-1 activates the proliferation of the ductal tree of the mammary gland [72]. IGF-1 is considered to be central to the process of ductal morphogenesis because neither estradiol (E2) nor progesterone (P) can act in the absence of IGF-1 [72]. Formation of the ductal tree is orchestrated by a specialized structure called the terminal end bud (TEB), which is responsible for the production of mature cell types leading to the elongation of the subtending duct. The TEB is also the regulatory control point for basement membrane deposition, branching, angiogenesis, and pattern formation [73]. It has been demonstrated in murine models that the earliest phase of pubertal mammary development (formation of TEBs) requires IGF-1. No other hormones have been shown to stimulate the formation of TEBs unless GH or IGF-1 is present. GH-induced IGF-1 is thus of major importance in ductal morphogenesis [74, 75]. It has been shown in the prepubertal mammary glands of BK5.IGF-1 transgenic (Tg) mice that IGF-1 preferentially activated the PI3K/AKT pathway via the formation of ERα/insulin receptor substrate 1 (IRS-1) complex [76]. Conversely, in postpubertal Tg glands, reduced ERα expression failed to stimulate the formation of the ERα/IRS-1 complex, allowing signaling to proceed via the alternate RAS/RAF/MAPK pathway [76]. Accordingly, changes in ERα expression at different stages of development direct IGF-1 signaling and the resulting tissue responses. As ERα levels are elevated during the prepubertal and postmenopausal stages, these may represent windows of susceptibility during which increased IGF-1 exposure maximally enhances BCa risk [76].

Elevations of plasma GH and IGF-1 concentrations by cow’s milk intake may thus enhance the physiological magnitude of circulating GH/IGF-1 during puberty deviating sebaceous gland, eye growth, and bone homeostasis (displaying acne, early-onset juvenile myopia, pubertal skeletal overgrowth) but indiscernibly disturbing mammary gland maturation and TEB formation potentially increasing the risk of BCa.

## Epidemiological Evidence

### Cow’s Milk Exposure in Pre- and Postmenopausal Women

There are controversial results with regard to the reported epidemiological study type, i.e., national cohort studies (retrospective versus prospective), case-control studies, and large multi-national meta-analyses including worldwide “umbrella” studies, concerning the outcomes of BCa risk among adult women consuming cow’s milk.

### Cohort Studies

According to the prospective study of the Norwegian Cancer Registry (*n* = 25,892), daily intake of > 750 ml whole cow’s milk compared to < 150 ml daily enhanced the risk of BCa by a factor of 2.91 [77]. A retrospective hospital-based case-control study (*n* = 1857 cases and 1202 controls) in the US found a positive association between cow’s milk intake and the risk of ER-negative BCa (OR: 1.58; 95% CI: 1.05–2.37) [78]. Fraser et al. [79••] recently reported an increase in BCa risk (HR = 1.50; 95% CI: 1.22–1.84) related to cow’s milk consumption independent from milk fat content in a Californian prospective cohort study of 52,795 women recruited from the Adventist Health Study-2, a large cohort of North American Adventists followed for 7.9 years. They found a stronger association between cow’s milk consumption with ER^+^ and PR^+^ tumors. The daily intake of 158 ml of milk already enhanced BCa risk, whereas the consumption of cheese and yogurt did not affect BCa risk [79••]. In contrast, Nilsson et al. [80] assessed the consumption of fermented milk, non-fermented milk, cheese, and butter, estimated from semiquantitative food frequency questionnaires, in relation to prospective risk of breast, prostate, colorectal, smoking-, and obesity-related cancers in 101,235 subjects, including 1921 BCa cases in the population-based Northern Sweden Health and Disease Study. They observed no consistent association between milk/dairy intake and BCa risk, whereas an increased BCa risk was observed for women in the third vs. lowest quintile of non-fermented milk intake [80]. Recently, Kaluza et al. [81••] presented the results of a population-based Swedish Mammography Cohort including 33,780 women (88.2% postmenopausal) and showed that high and continuous consumption of two daily servings of non-fermented milk compared to no milk consumption, significantly increased the incidence of ER^+^/PR^+^ BCa (HR = 1.30; 95% CI: 1.02–1.65).

These studies were derived from populations known for high dietary exposure to cow’s milk and dairy products and a high frequency of adult lactase persistence alleles thus allowing the consumption of higher quantities of milk compared to Asian populations, where lactase persistence is rare. A large cohort study including 22,788 subjects from the Swedish Cancer Registry with lactose intolerance (more common in individuals with an absence of lactase persistence, which is the ancestral human genetic trait [82]), and thus a lower milk and dairy intake, has found a lower risk of BCa (standardized incidence ratio = 0.79) [83].

Cow’s milk consumption was traditionally low in China (high genetic prevalence of lactase non-persistence). However, cow’s milk consumption in China increased due to governmental promotion. To investigate BCa risk factors in Chinese women residing in urban and rural areas of eastern China, a large-scale cross-sectional survey, which included 122,058 women, identified an increased risk of BCa associated with milk consumption in rural areas [84]. The prospective China Kadoorie Biobank Study recruited ~500,000 adults from ten diverse (five urban, five rural) areas across China during 2004–2008 with a mean follow-up of 10.8 years. A significant positive association was found between BCa and dairy consumption (predominantly milk) with an adjusted HR per 50 g/day consumption of 1.19 (95% CI: 1.01–1.41) (*n* = 2582) [85••].

### Case-control Studies

An Iranian population-based case-control study on 350 BCa patients and 700 age-matched controls found a positive association between total milk intake (OR 1.76; 95% CI: 1.16–2.65) and BCa, whereas no significant associations between yogurt and cheese consumption and BCa risk were observed [86•]. In contrast, a case-control study from Poland including 823 BCa cases using a semiquantitative food frequency questionnaire, which retrospectively evaluated the consumption of milk and dairy products for a time period of 10–15 years prior to BCa diagnosis, reported that high consumption of milk decreased the risk of BCa for both premenopausal and postmenopausal women [87]. Conversely, a case-control study in a Western Mexican population (97 BCa patients, 104 controls) showed that high milk consumption increased the BCa risk by 7.2 times [88]. Another case-control study in Uruguay reported that a high intake of whole milk was associated with a significant increased risk of BCa, whereas ricotta cheese and skim yogurt were associated with significant decreased risks [89].

### Meta-analyses Including International Studies

In 2015, Zang et al. [90] analyzed 22 prospective cohort studies (1,566,940 participants) and five case-control studies (33,372 participants) published between 1989 and 2013. High milk consumption was not found to have a preventive effect on BCa compared to low milk consumption (RR, 0.94; 95% CI: 0.86–1.03) and there was no evidence of a linear or nonlinear relationship between milk consumption and risk of BCa. The meta-analysis of Chen et al. [91] was reported in 2019 and selected data from 8 studies published between 1989 and 2009, and thus dismissed studies of a whole successive decade during which increasingly positive associations between milk intake and BCa risk have been reported [78, 79••, 81••, 85••, 86•, 88]. The authors concluded that all milk models and the “available epidemiologic evidence” do not support a strong association between the consumption of cow’s milk or milk products and BCa risk.

In 2021, He et al. [92]. performed a meta-analysis considering 36 articles with 1,019,232 participants including individuals of European and Asian populations. Although the authors did not present exclusive data for milk, non-fermented dairy product intake exhibited no significant associations for BCa in premenopausal (HR = 1.03; 95%CI: 0.97–1.09) and postmenopausal women (HR = 1.03; 95%CI: 0.98–1.08), respectively.

To evaluate the BCa risk of milk consumption during childhood and adolescence, Gil et al. [93] in 2022 provided summary RRs for the highest vs. lowest milk intake of 0.83 (95% CI: 0.69–1.00; *p* = 0.05; I2 = 60%) involving 6 studies published between 2001 and 2010, which relied on differing retrospective food questionnaires reporting milk consumption during childhood and adolescence and their potential risk association with BCa in adult life. One study reported on dietary habits in adolescence and midlife and the risk of BCa in postmenopausal women with a mean age of 77 years. Remarkably, milk intake during childhood and adolescence was evaluated retrospectively in these studies with nonuniform food questionaires which occurred in the 1950s, and thus recall of dietary behavior dated back for several decades pointing to serious limitations of this recently presented meta-analysis [93].

## Pathobiochemical Evidence

### Milk Components with Carcinogenic Potential

Several components in commercial cow’s milk may foster malignant transformation and may promote BCa initiation and progression. These factors are milk-derived and milk-induced IGF-1, estrogens, exosomal microRNAs, bovine meat and milk factors (BMMFs), and other contaminants like aflatoxins, bisphenol A, and micro- and nanoplastics as well as environmental pesticides [94•].

### Bovine Milk IGF-1

**Fig. 1:**
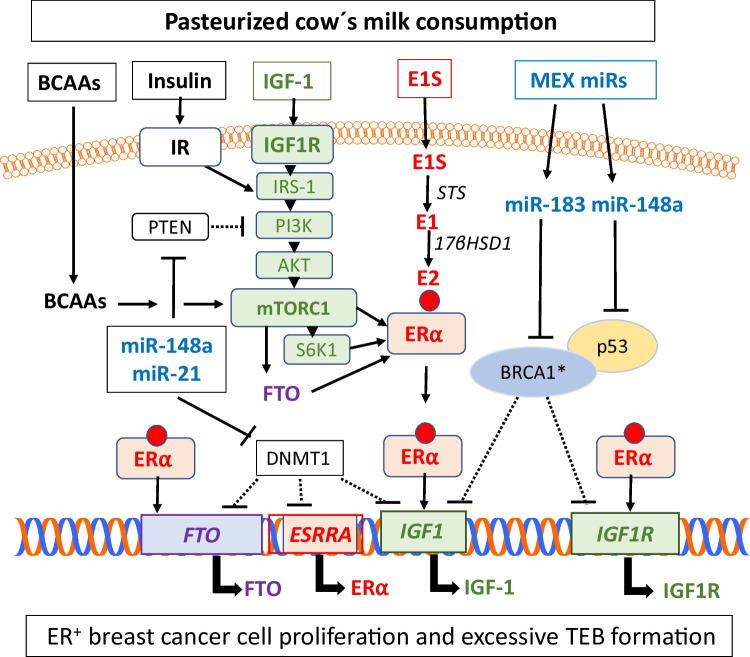
Synergistic interaction of milk-induced signaling pathways between branched-chain amino acids (BCAAs), insulin, insulin-like growth factor 1 (IGF-1), estrone sulfate (E1S), and milk exosome (MEX)–derived microRNAs (miRs) in breast cancer (BCa). Consumption of pasteurized cow’s milk increases circulatory levels of BCAAs, IGF-1, E1S, miR-148a, and miR-21 that may reach mammary gland epithelial and stromal cells. BCAAs and IGF-1 activate the mechanistic target of rapamycin complex 1 (mTORC1), which promotes the translation of estrogen receptor-*α* (ER*α*) and fat mass and obesity-associated gene (*FTO*). Phosphorylation of ER*α* by the kinase S6K1 and estradiol (E2) ligand binding activates ER*α* $,$ which stimulates the expression of IGF-1 and IGF-1 receptor (IGF1R). BCa cells are able to convert E1S to E2 via the action of steroid sulfatase (STS) and 17*β*-hydroxysteroid dehydrogenase type 1 (17 $\beta$ HSD1). Physiologically, BRCA1 via direct interaction with p53 inhibits gene expression of *IGF1* and *IGF1R*. However, loss-of-function mutations of BRCA1* enhance IGF-1 and IGF1R expression. MiR-148a signaling via suppression of p53 and DNA methyltransferase 1 (DNMT1) enhances the expression of IGF-1, ER*α*, and FTO, a m6A RNA demethylase, that further enhances ER*α* expression. In addition, E2 promotes FTO expression. Individuals with adipogenic SNPs of *FTO* may exhibit increased susceptibility for milk-mediated FTO expression. MEX miR-148a-induced suppression of p53 and MEX miR-183-mediated suppression of *BRCA1* may further enhance IGF-1 signaling in BCa

IGF-1 is a component of bovine milk and is not destroyed by pasteurization [95]. The natural concentration of IGF-1 in milk is 1.27–8.10 ng/ml [95], but IGF-1 concentration in 5777 random milk samples from Bavarian dairy cows ranged from 1.0 to 83.0 ng/ml [96]. According to a recent study in the US, median bovine GH and IGF-1 concentrations in conventional milk were 9.8 and 3.5 ng/ml, respectively, twenty and three times that found in organic samples [97]. Treatment of dairy cows with bovine somatotropin (bST) (forbidden in the European Union) to increase milk yield enhances IGF-1 concentrations in milk [96]. Notably, mastitis, a common inflammatory disease of lactating dairy cows, increases IGF-1 levels in milk whey [98]. Single-nucleotide polymorphisms (SNPs) of the bovine *IGF1* gene have been associated with milk yield [99]. IGF-1 is of pivotal importance for the maintenance of cow’s milk production during lactation and thus milk yield [100]. Accordingly, the breeding selection of dairy cows for the enhancement of lactation performance may have increased milk IGF-1 levels over decades. The highest milk IGF-1 content was observed for whole milk, followed by reduced-fat and low-fat milk, respectively [101], indicating that the milk fat fraction also contributes to total milk IGF-1 levels. However, it is not the bovine milk-derived IGF-1 that increases circulatory IGF-1 levels in human milk consumers but the milk-induced intrinsic synthesis and secretion of IGF-1 by the milk consumer [5, 7•] (Fig. 1).

### Commercial Milk Estrogens

Between 1850 and 1910, milk production was highly seasonal. Peak milk volumes occurred in the spring after calving and cows were not milked during the winter months [102]. Milk produced from “persistently” pregnant cows–the current routine praxis of the dairy industry–to increase commercial milk yield enhances milk estrogen concentrations. In pregnant cows, the predominant estrogen is estrone (E1) sulfate, which passes into milk. Heat treatment (70 °C and 95 °C) does not affect E1 and E2 concentrations compared to unprocessed raw milk [103]. The concentration of E1 sulfate increases from 30 pg/ml in non-pregnant cows to 151 pg/ml in pregnant cows at 40–60 days of gestation, and to a maximum level of 1000 pg/ml in cows at 220 days of gestation [104]. As milk of pregnant dairy cows is pooled, commercial milk contains higher estrogen amounts compared to former times, when lactation of cows was synchronized and cows gave birth only in spring time. Maruyama et al. [105] analyzed the exposure to exogenous estrogen through the intake of commercial milk produced from pregnant cows in children and adults. Urine concentrations of E1, E2, E3, and pregnanediol significantly increased in all adults and children after intake of 600 ml/m^2^ of commercial cow’s milk. In prepubertal children, urinary excretion volumes of estrogens and pregnanediol significantly increased within 1–3 h. The net increase of E2 excretion from the basal E2 levels in urine (before the intake) was 39–109 ng/4 h in this study. These data indicate that the intake of estrogens from cow’s milk corresponds to the daily estrogen production rate in prepubertal boys. Maruyama et al. [105] concluded that the intake of cow’s milk may be one of the major causes of early sexual maturation in prepubertal children. Peaker of the Hannah Dairy Research Foundation [106] did not consider these pediatric concerns [105] and stated that “even in worst case scenarios, oestrogen consumption in milk is considerably less than regulatory bodies regard as entirely safe.” Nevertheless, BCa cells are able to convert E1 sulfate into the 10 times more biologically active E2 [107]. Additionally, it is well accepted that even low doses of estrogens can both initiate as well as promote the growth of existing BCa [108, 109].

Furthermore, the molecular crosstalk between IGF-1 and E2 has potentiating synergistic effects in breast carcinogenesis. IGF-1 is a key activator of mTORC1 [5, 7•]. mTORC1 emerged as a critical node in estrogenic signaling in BCa cells. Estrogens rapidly and potently activate mTORC1, which is a crucial activator of ER*α* transcriptional activity [110]. mTORC1 and its downstream kinase S6K1 directly phosphorylate and activate ERα upon estrogen stimulation, which implicates activated mTORC1 signaling in the pathogenesis of ER^+^ BCa [111, 112]. There is a close interaction between ERα and insulin/IGF signaling in BCa [113, 114]. IGF-1 and IGF-2 are among the most potent mitogens for mammary epithelial cells and there is accumulating evidence that they interact with the E2 axis to regulate mitogenesis, apoptosis, adhesion, migration, and differentiation of mammary epithelial cells. Such interactions are bi-directional and E2 has been shown to regulate the expression and activity of IGF genes with the general effect of sensitizing breast epithelial cells to the actions of IGFs and insulin [113].

Samoli et al. [115] provided evidence in support of an interaction of IGF-1 with the expression of ERα in the non-malignant mammary tissue in the context of BCa pathogenesis. As shown in ovarian cancer cell lines, E2 induces gene expression of IGF-1 and c-myc and increases the binding of ERα to the promoters of IGF-1 and c-myc [116]. The activation of ERα in BCa upregulates the expression of IGF-1, IRS-1, and IGF1R, which results in the amplification of IGF-1 responses. Reciprocally, stimulation of IGF1R increases the phosphorylation and activity of ERα [117]. In ER^+^ BCa cells (MCF-7 cells), E2 treatment significantly activated the *IGF1R* promoter [118] (Fig. 1). Unfortunately, IGF1R expression is not controlled in the routine pathology of BCa.

The association between fat mass and obesity (*FTO*) gene polymorphisms and BCa is influenced by the status of ERs. Estrogens may promote BCa cell proliferation through upregulation of *FTO* gene expression and activation of the PI3K/AKT signaling pathway in ER^+^ BCa patients [119•]. Translational evidence indicates that milk intake enhances FTO-mediated transcription [120]. There appears to be a significant difference in the magnitude of *FTO* induction between human and bovine milk [120].

### Impact of BRCA1 on IGF-1/IGF1R Signaling

*BRCA1*, the well-established susceptibility gene for BCa and ovarian cancer [121], increases intratumoral IGF-1 protein expression in *BRCA* mutation carriers suggesting an involvement of the IGF-1/IGF1R axis in BCa pathogenesis [122]. Kang et al. [123] showed that in human BCa cells, *IGF1* gene expression is negatively regulated by BRCA1 at the transcriptional level. The *IGF1R* gene is also under negative control by BRCA1 and p53, which both physically interact with the *IGF1R* promoter [124] (Fig. 1). Thus, the loss of BRCA1 function can overstimulate IGF-1/IGF1R/PI3K/AKT/mTORC1 signaling, which significantly contributes to an increase in cell survival and proliferation thus promoting BCa. Elevated circulatory IGF-1 levels via milk consumption may thus have potentiating effects on the initiation and promotion of BCa. In fact, a recent observational and Mendelian randomization study confirmed a causal role of circulatory IGF-1 in BCa [125•].

### Cow Milk’s Exosomal MicroRNAs with Oncogenic Activity

Cow´s milk contains abundant exosomes (nanoparticles of ~100 nm diameter) carrying microRNAs (miRs) that survive pasteurization but not ultra-heat treatment (UHT) of milk [126•, 127•]. Abundant signature miRs of pasteurized cow’s milk, milk fat, and milk exosomes (MEX) are bovine miR-148a-3p and miR-21-5p [128, 129]. These share identical nucleotide sequences with the corresponding human miRs. Notably, *MIR148A* is a domestication gene of dairy cattle, which increases milk yield [130, 131]. After oral administration, bovine MEX stays bioavailable and reach the systemic circulation and the tissues of mice and humans [132•, 133•, 134•]. MEX miR-148a-3p targets the mRNA of DNA methyltransferase 1 (*DNMT1*) [129], thereby enhancing the expression of ERα in BCa cells [135] and stimulating IGF-1 expression [136]. In addition, MEX miR-148a-3p attenuates the expression of the tumor suppressor p53 [137•], which is a negative transcriptional regulator of *IGF1R* [138, 139] (Fig. 1). Accordingly, increased serum levels of miR-148a-3p in humans were positively correlated with the presence of BCa [140•].

Regarding miR-21-5p, it is overexpressed in BCa compared with normal breast tissue and can promote the proliferation and invasion of BCa cell lines, and suppresses its downstream target gene phosphatase and tensin homolog (PTEN) [141] (Fig. 1). Via suppressing PTEN, miR-148a-3p and miR-21-5p enhance PI3K/AKT/mTORC1 signaling. Interestingly, labeled bovine MEX and miR-21-5p accumulate in murine placenta and embryos following oral gavage [133•]. Notably, increased levels of miR-21-5p have been detected in the placenta of infants with increased birthweight [142, 143]. Additionally, placental miR-21 overexpression is associated with increased fetal growth [144•, 145]. Thus, bovine MEX miR signaling during pregnancy via cow’s milk intake may also affect fetal mammary gland growth trajectories.

MiR-21-5p is significantly upregulated in BCa tissue, cells, and exosomes [146•]. Notably, after the consumption of 1 L of commercial 1% fat cow’s milk, miR-21-5p plasma levels significantly increased by 147% in human volunteers for 3.2 h postprandially [134•]. Of importance, mastitis, a common inflammatory complication of lactating dairy cows, is associated with enhanced expression of miR-21 in the milk of affected cows [147], thus increasing the load of this oncogenic miR in milk [148].

Cancer cell metabolism is characterized by a shift from an oxidative to a glycolytic bioenergetic pathway, a process known as the Warburg effect. Both miR-148a-3p and miR-21-5p promote hypoxia-inducible factor (HIF)–induced glycolysis via targeting hypoxia-inducible factor 1α inhibitor (*HIF1AN*) and von Hippel-Lindau tumor suppressor (*VHL*), respectively. Of note, HIF-dependent hallmarks of cancer include angiogenesis and metabolic rewiring, which are well-established drivers of BCa aggressiveness, therapy resistance, and poor prognosis [149]. Furthermore, it has been reported in BCa that overexpression of miR-378* suppresses estrogen-related receptor-*γ* (*ESRRG*) [150], a key transcription factor regulating mitochondrial oxidative phosphorylation. *ESRRG* is also a target gene of miR-148a-3p (targetscan.org), which may thus synergize with miR-378* in promoting glycolysis-induced BCa cell proliferation [150]. Thus, bioactive oncogenic miRs of cow’s milk that survive pasteurization and are increased by breeding selection and bovine mastitis may epigenetically promote BCa carcinogenesis. Figure 1 illustrates the potential molecular crosstalk of milk-induced IGF-1, estrogen, and miR signaling in the pathogenesis of BCa. Of importance, MEX miR-183, which is overexpressed in the milk of cows with *Staphylococcus aureus*–induced mastitis [151], has been shown to aggravate BCa [152•, 153, 154, 155•]. Furthermore, *BRCA1* is a predicted target gene of miR-183-5p.2 (targetscan.org). MiR-183-5p via targeting *BRCA1* may thus increase IGF-1 expression, which is in fact upregulated in the milk of cows suffering from mastitis [98].

### Bovine Meat and Milk Factors

Uptake of dairy products of *Bos taurus*–derived dairy cows, particularly consumed at an early age, is suggested to represent one of the main risk factors for the development of BCa [156]. Virus-like circular DNAs of bovine meat and milk factors (BMMFs) have been recently isolated from commercial milk [157] and BCa tissue [158•]. Transcriptome profiling upon BMMF expression identified host cellular gene expression changes related to cell cycle progression pointing to a potential pathogenic involvement of BMMFs in BCa [159•]. Interestingly, certain oncogenic viruses operate via the activation of IGF-1 signaling [160].

### Milk Aflatoxins

Ruminants hydroxylate aflatoxin B1 (AFB1), ingested by contaminated food, to aflatoxin M1 (AFM1), which is excreted into cow’s milk [161]. AFM1 in milk is among the most carcinogenic compounds, especially when relatively high levels are consumed in vulnerable age groups, i.e., infants and the elderly [161]. The increase of AFM1 concentrations in the milk of maize-fed cows due to climate change is a recent matter of concern [162•]. Concentrations of AFB1 and AFM1 of pasteurized and UHT milk were detected in the range of 0.7–1.5 μg/l [163]. High concentrations of AFM1 have recently been detected in human breast milk [164]. As shown in hepatocytes, AFB1 stimulates PI3K/AKT signaling [165] and may thus converge with milk-induced IGF-1/PI3K/AKT signaling.

### Milk Contamination with Bisphenol A

Recently, the endocrine disruptor bisphenol A (BPA), a synthetic compound with estrogenic activity, has been detected in raw and processed milk [166•, 167•]. The average concentration of bisphenol A found in milk from cartons (0.87 ng/ml) was greater than in milk from plastic bottles (0.35 ng/ml) [168•]. The maximal probable daily intake (PDI) of BPA by (ng/kg per capita) by milk consumption has been presented for Norway (0.73), Austria (3.21), France (7.75), Italy (7.87), and Belgium (15.16), respectively [169]. These PDI values are in the range for other BPA-contaminated foodstuffs like beverages, seafood, and meat [169]. A recent Nigerian study showed that in the category of dairy products, the highest daily intake of BPA was in canned evaporated milk, followed by packaged cheese while raw cheese did not contribute to the total estimated daily intake [170]. Thus, milk consumption significantly contributes to the total estimated daily dietary BPA intake. This is relevant, because even low-dose BPA exposure is a matter of concern for BCa development and may affect vulnerable periods of breast development [171]. In mice, perinatal exposure to BPA increased the number of TEBs and the progesterone response of mammary epithelial cells [172]. In rats, perinatal exposure to BPA induced ductal hyperplasia, ductal carcinoma in situ, and malignant tumors [173, 174]. In rhesus monkeys, fetal exposure to BPA increased the density of mammary buds and accelerated mammary epithelial development [175]. In ovarian cancer cells, BPA enhanced the crosstalk between ERα and IGF1R signaling pathways [176]. Thus, milk-derived estrogens and BPA in concert with further dietary BPA exposure may have potentiating effects in BCa carcinogenesis.

### Milk Contamination with Xenobiotics, Pesticides, Microplastics, and Nanoplastics

Pesticides are among the most commonly found contaminants, not only in raw cow’s milk but also after milk pasteurization and UHT processing [94•]. Oxidative stress caused by pesticides is an important mechanism through which pesticides exert their harmful effects. Many pesticides have been shown to modulate gene expression at the level of non-coding RNAs, histone deacetylases, and DNA methylation patterns suggesting their role in epigenetic deviations [177]. It is impossible to oversee the impact and interaction of all pesticide and xenobiotic contaminations in commercial milk that may as well modulate the GH-IGF axis [178].

Humans are estimated to ingest tens of thousands to millions of microplastic (MP) particles annually, in the order of several milligrams daily [179]. Available information suggests that inhalation of indoor air and ingestion of drinking water bottled in plastic are the major sources of MP exposure. However, little is known about dietary MP exposure in humans. In newborns and infants, bottle feeding and medical devices can contribute to MP ingestion [179]. The detection of MP in seafood, honey, milk, beer, table salt, drinking water, and air is a recent matter of concern [180]. The concentration of MPs identified in cow´s milk samples ranged from 204 to 1004 MPs per 100 ml exhibiting a surface area mainly ≤ 50 μm^2^ [181•]. The increased paraben-induced proliferation of estrogen-sensitive BCa cells was augmented in the presence of plastic nanoparticles. The mechanism may be related to the translocation and adsorption properties of nanoplastics acting as a Trojan horse to expose cells to parabens more efficiently [182•]. The frequently used microplastic-derived plasticizer organophosphate ester tri-o-cresyl phosphate interacts with ERα in MCF-7 BCa cells promoting cancer cell growth [183•].

Table 1 summarizes the spectrum of potential milk-derived carcinogenic compounds that may vary in relation to many genetic and environmental factors.

**Table 1:** Milk-derived potential carcinogenic compounds that vary in relation to genetic and environmental factors of dairy cows, milk processing and distribution

**Carcinogenic factors**	**Potential effects on breast carcinogenesis**
Dairy cow selection by *IGF1* gene polymorphisms associated with increased lactation performance	Increased milk levels of IGF-1, which enhances mitogenic IGF-1 signaling
Treatment of dairy cows with bovine somatotropin (bST) to increase milk yield	Increased milk levels of mitogenic IGF-1
Modification of milk compounds with oncogenic potential by infectious mastitis	Increased concentrations of IGF-1, miR-21 and miR-183 in milk thus promoting IGF-1/AKT/mTORC1 signaling
Whole milk compared to skim milk	Higher milk levels of IGF-1 in whole milk
Increased *MIR148A* expression increasing milk yield	Increased levels of miR-148a-3p in milk enhancing PI3K/AKT/mTORC1 signaling, glycolysis, and IGF-1 and ER*α* expression via suppression of DNMT1
Dairy cow strains transfected with BMMFs	Stimulatory effects on cell proliferation
Increased levels of aflatoxin M1 (AFM1) in maize-fed cows compared to grass-fed cows	Synergistic effects of AFM1 on PI3K/AKT/mTORC1 signaling
Milk contamination with bisphenol A (BPA)	Synergistic effect of BPA on mitogenic IGF-1 and ER*α* signaling
Milk microplastics and nanoplastics	Potential catalytic action on estrogen signaling
Variations in thermal milk processing	Increased survival of oncogenic exosomal miRs by pasteurization compared to cooking and ultra-heat treatment (UHT)
Variations in microbial milk processing	Bioactivity of exosomal miRs in non-fermented pasteurized milk compared to decreased bioactivity of oncogenic exosomal miRs by microbial fermentation

## Conclusions

Two recent prospective cohort studies in California [79••] and Sweden [81••] identified cow’s milk consumption as a nutritional risk factor for ER^+^ BCa in North American and European populations. Epidemiological evidence, albeit conflicting, is supported by pathobiochemical pathways of milk-derived signaling (Fig. 1). The molecular crosstalk between BRCA1, FTO, IGF-1, IGF1R, E2, MEX miRs, AFM1, BMMFs, BPA, and MP/nanoplastics may potentiate oncogenic signal transduction pathways promoting BCa initiation and progression. According to NHANES III, adult IGF-1 levels and IGF-1/IGFBP-3 molar ratio had significant inverse associations with adolescent milk intake in non-Hispanic White men, but not in men of other ethnicities or in women [184•]. The sequential impact of cow’s milk signaling during vulnerable periods of human life in the pathogenesis of BCa has never been investigated by epidemiological research. The majority of epidemiological studies focus on pre- and/or postmenopausal women in relation to retrospectively estimated milk consumption missing the most critical periods of breast morphogenesis during fetal and puberty-associated breast tissue development and TEB formation (Table 2).

**Table 2:** Ethnic, individual and environmental factors modifying the susceptibility to milk’s nutrigenomic effects

**Genetic and environmental predisposition factors of milk consumers**	**Nutrigenomic effects in milk consumers enhancing breast carcinogenesis**
Lactase (*LCT*) persistence	Tolerance to higher quantities of cow’s milk intake compared to individuals with lactase non-persistence (Asian populations)
*IGF1* single nucleotide polymorphisms with enhanced IGF-1 expression	Genetically predisposed IGF-1 hyper-responders may be at increased risk for milk-induced IGF-1 signal transduction
Variations in the prevalence of *BRCA1* loss-of-function mutations	Enhanced IGF-1 signaling compared to ethnic groups with lower prevalence of *BRCA1* mutations
Variations in the frequency of adipogenic *FTO* gene polymorphism	Synergism of milk-derived estrogen exposure and FTO-mediated estrogen signaling
Molecular heterogeneity of hormone receptor expression (ER^+^, ER^−^, PR^+^, PR^−^, HER2^+^, HER2^−^, triple negative BCa)	Milk signaling may preferentially promote ER-positive BCa
Variations in cow’s milk exposure during vulnerable periods of breast development, mammary branching morphogenesis, and tubular end bud formation	Enhanced risk of breast carcinogenesis by lifetime milk exposure including pregnancy (fetal overgrowth, increased birthweight), childhood and puberty (early menarche, increased longitudinal bone length), and pre- and postmenopausal periods
Variations in the dietary intake of high glycemic carbohydrates in combination with milk (milk + sugar); variations in the prevalence of type 2 diabetes	Western populations exposed to high glycemic load diets exhibit increased serum insulin and IGF-1 levels; diabetes type 2 is associated with increased risk of BCa
Quantitative and qualitative variations in protein intake	High milk protein (yogurt, cheese) and animal protein (meat) intake further increase serum IGF-1 concentrations
Variations in frequency and duration of artificial estrogen administration	Synergism of milk-induced and iatrogenic estrogen exposure enhancing estrogen and IGF-1 signaling
Variations in breastfeeding versus artificial formula feeding	Artificial formula feeding during the postnatal period may deviate postnatal breast morphogenesis via increased FTO expression
Variations in the mode and duration of breastfeeding	Reduced risk of BCa by prolonged breastfeeding of the offspring
Maternal variations in the duration of breastfeeding	Reduced risk of BCa in mothers who offer prolonged breastfeeding to their infants

Milk is a highly complex bioactive fluid containing multiple biological and environmental effectors and thus is not a suitable single variable for epidemiological studies like blood pressure. Most published meta-analyses ignore the processing of milk, especially the thermal effects of pasteurization versus UHT, and thus do not provide information on the presence or absence of oncogenic MEX miRs [185]. Genetic variations of domestic cows like SNPs of the bovine *IGF1* gene, breeding selections for lactation performance (enhanced bovine *MIR148A* expression), prevalence of mastitis, feeding procedures (grass versus corn), and environmental contaminations (aflatoxins, bisphenol A, pesticides, BMMFs, MPs, nanoplastics) are not and could never be taken into account by epidemiological studies, but may all have synergistic impacts on milk’s oncogenic signaling capacity (Table 1).

On the side of the milk consumer, there is as well a great genetic and nutrigenomic heterogeneity. Genetic susceptibility explains 5–10% of all BCa cases. *BRCA1* and *BRCA2* genes are the most common cause of hereditary BCa exhibiting ethnic differences [186•]. A recent multicenter study comprehensively describes the characteristics of the 1650 unique *BRCA1* and 1731 unique *BRCA2* deleterious (disease-associated) mutations identified in the CIMBA database and observed substantial variation in mutation type and frequency by geographical region and race/ethnicity [187••]. Women with *BRCA1* loss-of-function mutations in milk-consuming Western societies may thus exhibit increased susceptibility to milk-derived IGF-1 signaling as mutant BRCA1 exhibits less inhibitory activity on IGF-1 and IGF1R expression [123, 124]. Furthermore, women with certain *FTO* and *IGF1* gain-of-function gene polymorphisms appear to be at increased risk for BCa [26–28, 188, 189] (Table 2).

There is mounting epidemiological evidence of a robust association between type 2 diabetes (T2D) and an increased risk of common cancers including BCa [190•]. Current understanding of the complex signaling pathways underlying the obesity/T2D/BCa link focuses particularly on the insulin/IGF system [191, 192]. In fact, milk-induced insulin, IGF-1, and MEX miR-148a-3p/miR-21-5p-mediated signaling pathways have recently been linked to the pathogenesis of T2D [7•, 193, 194••, 195, 196] relating milk consumption to the pathogenesis of both T2D and BCa.

Large meta-analyses include populations of different ethnic origins like Europeans exhibiting lactase persistence (allowing high amounts of milk intake) and Asian populations with lactase non-persistence (naturally restricting milk consumption). We conclude that multi-national meta-analyses intended to present extremely high proband numbers are less reliable for an adequate estimation of BCa risk as they do not consider the genetic heterogeneity of BCa patients, i.e., prevalence of germline and somatic mutations in a milk-consuming population. In contrast, prospective studies including subjects derived from circumscribed homogenous ethnic populations with comparable genetic backgrounds as well as dairy cows of a given genetic background offer a more realistic evaluation of the potential carcinogenic effects of cow’s milk consumption.

In summary, available data from recent prospective cohort studies as well as pathobiochemical insights into synergies between milk and BCa signaling pathways identify commercial cow’s milk consumption as a critical risk factor for ER-positive BCa in close analogy with milk’s impact on the pathogenesis of prostate cancer, the most common cancer in men [197–199].

In our opinion, current guidelines for dairy milk consumption should be viewed with caution. They do not appropriately differentiate between the biological effects of pasteurized milk, UHT milk, and fermented dairy products [6]. They neglect the signaling effects induced by cow’s milk consumption during vulnerable periods on human mammary morphogenesis, especially during pregnancy [200], childhood and puberty (school milk programs) [201], and adulthood like US Dietary Guidelines 2015–2020 recommending a daily intake of ~ 300 ml of cow’s milk [202]. Even, the updated Dietary Guidelines for Americans 2020–2025 still recommend fat-free and low-fat milk as core elements of a healthy diet [203]. In contrast, Fraser et al. [79••] observed a significantly increased risk of BCa in US women by daily intake of ~ 160 ml milk independent of milk’s fat level. In accordance with Wehbe and Kreydiyyeh [204], it is time to reconsider dairy recommendations, especially milk intake.

## Data Availability

This article is distributed under the terms of the Creative Commons Attribution 4.0 International License (http://creativecommons.org/licenses/by/4.0/), which permits unrestricted use, distribution, and reproduction in any medium, provided you give appropriate credit to the original author(s) and the source, provide a link to the Creative Commons license, and indicate if changes were made.
